# Caspase-11 signaling promotes damage to hippocampal CA3 to enhance cognitive dysfunction in infection

**DOI:** 10.1186/s10020-024-00891-y

**Published:** 2024-08-23

**Authors:** Ni Liang, Yi Li, Chuang Yuan, Xiaoli Zhong, Yanliang Yang, Fang Liang, Kai Zhao, Fangfang Yuan, Jian Shi, Erhua Wang, Yanjun Zhong, Guixiang Tian, Ben Lu, Yiting Tang

**Affiliations:** 1 Department of Physiology, School of Basic Medical Science, Central South University, Changsha, 410000 People’s Republic of China; 2 Department of Critical Care Medicine and Hematology, The 3Rd Xiangya Hospital, Central South University, Changsha, 410000 People’s Republic of China; 3 Xiangya School of Pharmaceutical Sciences, Central South University, Changsha, 410000 People’s Republic of China; 4 ICU Center, The Second Xiangya Hospital, Central South University, Changsha, 410000 People’s Republic of China; 5 Department of Ultrasound, The Second Xiangya Hospital of Central South University, Changsha, 410000 People’s Republic of China; 6 Hunan Key Laboratory of Organ Fibrosis, Xiangya Hospital, Central South University, Changsha, 410000 People’s Republic of China; 7 Key Laboratory of Sepsis Translational Medicine of Hunan, Central South University, Changsha, Hunan Province 410000 People’s Republic of China

**Keywords:** Endotoxemia, Cognitive dysfunction, Neuroinflammation, Casp11, GSDMD, Blood‒brain barrier, Microglia, CA3

## Abstract

**Background:**

Cognitive dysfunction caused by infection frequently emerges as a complication in sepsis survivor patients. However, a comprehensive understanding of its pathogenesis remains elusive.

**Methods:**

In our in vivo experiments, an animal model of endotoxemia was employed, utilizing the Novel Object Recognition Test and Morris Water Maze Test to assess cognitive function. Various techniques, including immunofluorescent staining, Western blotting, blood‒brain barrier permeability assessment, Limulus Amebocyte Lysate (LAL) assay, and Proximity-ligation assay, were employed to identify brain pathological injury and neuroinflammation. To discern the role of Caspase-11 (Casp11) in hematopoietic or non-hematopoietic cells in endotoxemia-induced cognitive decline, bone marrow chimeras were generated through bone marrow transplantation (BMT) using wild-type (WT) and Casp11-deficient mice. In vitro studies involved treating BV2 cells with E. coli-derived outer membrane vesicles to mimic in vivo conditions.

**Results:**

Our findings indicate that the deficiency of Casp11-GSDMD signaling pathways reverses infection-induced cognitive dysfunction. Moreover, cognitive dysfunction can be ameliorated by blocking the IL-1 effect. Mechanistically, the absence of Casp11 signaling significantly mitigated blood‒brain barrier leakage, microglial activation, and synaptic damage in the hippocampal CA3 region, ultimately leading to improved cognitive function.

**Conclusion:**

This study unveils the crucial contribution of Casp11 and GSDMD to cognitive impairments and spatial memory loss in a murine sepsis model. Targeting Casp11 signaling emerges as a promising strategy for preventing or treating cognitive dysfunction in patients with severe infections.

## Background

Severe infection leads to sepsis, which has a high mortality rate and complex pathogenesis (Singer et al. 2016). Patients are often admitted to the intensive care unit due to severe infection, and nearly half of them exhibit encephalopathy symptoms such as altered consciousness, which can further increase their mortality. Even if some patients survived from survive sepsis, they will also have persistent cognitive impairment (Iwashyna et al. 2010; Barichello et al. 2019). Cognitive impairment resulting from infection represents a significant clinical challenge requiring resolution. However, the complete understanding of its pathogenesis remains elusive.

Endotoxin, or lipopolysaccharide (LPS), serves as the primary pathogenic component in the cell wall of gram-negative bacteria. In 1998, Toll-like receptor 4 (TLR4) was identified as the cell surface receptor for LPS (Poltorak et al. 1998). In recent years, researchers have discovered the receptor of intracellular LPS in mice, namely Caspase-11 (Casp11) (Kayagaki et al. 2011, 2013; Shi et al. 2014). Gasdermin D (GSDMD) is a protein that is activated by Casp11 and then undergoes self-shear to become the N-terminal and C-terminal regions. The N-terminus undergoes oligomerization to form holes in the cell membrane (Shi et al. 2015; Ding et al. 2016). These GSDMD pores not only changes the internal and external osmotic pressure of cells, leading to cell swelling and lysis, but also releases a large number of inflammatory mediators, such as IL-1α and IL-1β, which cause severe inflammatory effects (Broz and Dixit 2016; Galluzzi et al. 2018; Rathinam et al. 2019). Recently, it was reported that living cells can release inflammatory mediators such as IL-1 through GSDMD pores without pyroptosis (Evavold et al. 2018).

Activated microglia have been recognized as pivotal contributors to the onset of cognitive impairment induced by severe infection (Zrzavy et al. 2019). Furthermore, MRI examinations in sepsis patients have revealed vasogenic edema and white matter hyperintensities associated with blood‒brain barrier (BBB) damage (Ehler et al. 2017). Notably, the hippocampus, intricately linked to learning and memory functions, stands out as a focal area of interest (Barbizet 1963; Kimble and Pribram 1963). The hippocampal CA3 region is important for the rapid encoding of memory that arises from different spatial functions (O'Keefe and Dostrovsky 1971). However, how they are linked to infection-induced cognitive dysfunction remains unclear.

Addressing the significant clinical issue of infection-induced cognitive impairment, we employed a mouse model of endotoxemia. This study aims to uncover the role and mechanism of Casp11-GSDMD signaling specifically in infection-related cognitive impairment.

## Methods and materials

### Animal model

The mice of B6.SJL and C57BL/6 were purchased from Hunan SJA Company. Gsdmd-KO mice were donated by Academician Jiahuai Han (School of Life Sciences, Xiamen University) (He et al. 2015). Il-1r-KO mice were purchased from the Jackson Laboratory, while Casp11-KO mice were generously provided by Professor Timothy R. Billiar (University of Pittsburgh Medical Center) (Deng et al. 2018). The average body weight of the mice was 25 to 30 g and aged 8–12 weeks unless specified otherwise. The mice were raised in a specific pathogen-free conditions in the Department of Laboratory Animals of Central South University. Standard conditions included a 12 h light–dark cycle and a temperature range of 22–25 °C. The animal model uses intraperitoneal injection of 8 mg/kg LPS (from Escherichia coli (O111:B4), Sigma L2630) to induce cognitive disorder.

### Novel object recognition test

On Day 5 post-LPS challenge, we conducted a Novel Object Recognition (NOR) test in a 40 × 40 cm field arena to evaluate the mice's ability to discriminate a novel object. The test comprised two main phases: training and testing (Antunes and Biala 2012). In the training phase, we used two identical objects and symmetrically placed on the diagonal of the arena. Each mouse, positioned in one corner facing the wall, was given 10 min of free exploration while being recorded with the Smart system. To prevent olfactory cues, the arena was thoroughly cleaned with 75% alcohol between each test. After a 24 h interval, the testing phase commenced. We replaced the previous object with a new object and the mice's preference for the new object reflected the mice's desire to explore. Mouse movement recording and data analysis were using the VisuTrack system (Shanghai XinRuan Information Technology Co., Ltd). The discrimination index is calculated as the ratio of time spent with the new novel object to the total time with both objects, served as the metric for evaluating discrimination ability. All behavioral experiment operations and data analyses were conducted in a blinded, randomized fashion.

### Morris water maze test

The Morris Water Maze test, conducted from Day 7 to Day 11 post-LPS challenge, assessed spatial learning and memory in mice (Morris 1984). Mouse movements recording and data analysis were using the Smart3.0 system (Panlab Harvard Apparatus). The test employed a circular water tank, 120 cm in diameter, divided into four quadrants, each marked by distinct visual cues on the pool wall. An escape platform, hide in 1 cm below the water’s surface and concealed with edible titanium powder, was placed in one quadrant. The temperature of water was maintained at approximately 20 °C. Prior to the formal experiments, mice were acclimated to the water environment by spending 30 s on the platform. Subsequently, mice were introduced into the water from different entry points, facing the pool wall. Over four days, mice underwent training sessions three times a day, with a 1 min time limit to locate the platform. Mice failing to reach the platform within the allotted time were guided to it and allowed a 10 s stay; the latency to the platform was recorded. On the fifth day of the test period, we removed the platform and released the mice from a quadrant opposite the platform location into the water for 60 s. Spatial cognitive ability and memory were assessed through indicators such as the frequency of platform crossings, the latency to first reach the platform, and the proportion of time spent by mice in the target quadrant.

### Western blot analysis

The separated proteins were using 10% or 12% sodium dodecyl sulfate‒polyacrylamide gel electrophoresis and subsequently transferred onto 0.22 μm PVDF membranes (Millipore). Following a 1 h room temperature blockade with 10% nonfat dry milk in TBST buffer, PVDF membranes were incubated with primary antibodies overnight at 4 °C. Detailed antibody information is as follows: IBA1 (Wako#019–19741, 1:1000), Casp11 (Novusbio, NB120-10454, 1:200), GSDMD (Abcam, ab209845, 1:1000), β-actin (Cell Signaling Technology, 1:5000). The second antibody (Jackson, 1:5000) was incubated at room temperature for 1 h. Western Bright ECL Spray was employed for band visualization.

### Immunofluorescent staining

All animals underwent perfusion from the left ventricle with 20 ml PBS at 4 °C followed by 4% PFA (Paraformaldehyde). The entire brains, preserved in 4% PFA overnight, were subsequently removed. Dehydration was carried out in incrementing sucrose concentrations (15%, 25%, 35%, dissolved with PBS), and the brains were embedded in OCT, snap-frozen at -80 °C, and 20μm thick coronal sections were prepared at −20 °C low temperature thermostat. Following a 1 h 25 ℃ blocked with 0.1% Triton X-100 and 5% BSA, sliced brain was incubated with primary antibodies overnight at 4 °C: Ionized calcium-binding adaptor molecule 1 (Wako#019–19741, IBA1, 1:500), PSD-95 (Thermo Fisher Scientific#51–6900, 1:200,), Synaptophysin (Sigma#S5768, 1:200). Subsequently, sections underwent a 2 h incubation with secondary antibodies (Thermo Fisher Scientific#A10521 and F-2765, 1:500). Washed with PBST (0.01 M PBS containing 0.1% Triton X-100) for three times. Visualization and quantification by using Nikon Ni-U microscope and Image-J software. Confocal images were obtained using the Zeiss LSM800 confocal microscope.

### Blood brain barrier permeability

The experiment of blood brain barrier permeability was assessed using Evans blue extravasation (Sigma E2129). A 2% solution of Evans blue (diluted in saline, 80 ml/kg) was intravenously injected through the medial canthus vein immediately after the LPS challenge, and circulation was allowed for 2 h. Then, the brains were perfused with 20 ml PBS and removed on a background of paper to take gross photographs. Brain samples were weighed to determine the wet weight and fixed using dimethylformamide (dimethylformamide: brain wet weight = 1000 μl: 300 mg). Following homogenization, incubation at 37 °C for 24 h, and subsequent centrifugation, fluorescence levels were measured with a microplate reader (emission at 680 nm and excitation at 620 nm). The Evans blue in tissue content was quantified using a linear standard curve derived from known dye amounts. For fluorescent panoramic scanning, images of 30 μm thick coronal sections post-Evans blue challenge were captured using a Keyence BZ-X microscope.

### Purification of bacterial OMVs

We purified Outer Membrane Vesicles (OMVs) with E. coli BL21, the method following a previously described method with some modifications (Chutkan et al. 2013). Briefly, E. coli BL21 were cultured in an appropriate amount of LB solution until reaching an OD600 of ~ 0.5. To obtain the sterile supernatant, we first centrifuge the bacterial solution from the ice bath at 10,000 × g for 10 min at 4 °C. Then, the supernatant underwent further filtration through a 0.45 µm filter (Millipore), followed centrifugation at 10,000 × g for 10 min at 4 °C for the second time and filtration through a 0.22 µm filter (Millipore). OMVs were then obtained by ultracentrifugation at ~ 100,000 × g for 2 h at 4 °C using a Beckman Ti70 rotor. We removed the supernatant, the OMVs were resuspended in 500 µl sterile Dulbecco's Phosphate-Buffered Saline (DPBS) (Gibco) and filtered through a 0.22 µm filter. Purified OMVs underwent agar plating to confirm the absence of bacterial contamination, using a BCA protein assay kit (Thermo Scientific) to determine the protein content of OMV.

### Cell culture and stimulations

1 × 10^6 BV2 cells were treated with OMVs for 18h at indicated doses or left untreated in a 6-well plate. Then the supernatants were collected 18h post-stimulation to test cell death. For in vitro siRNA silencing of Casp11, BV2 cells were cultured in 6-well plates at a density of 0.2 million cells/well for transfection. We utilized Lipofectamine RNAi MAX Transfection Reagent (Invitrogen; 13778150) for siRNA transfection. Cells were stimulated with OMVs after siRNA transfection 48 h. The siRNA target sequences were CCUGAAGAGUUCACAAGGCUUTT (mCasp11) and UUCUCCGAACGUGUCACGUTT (control). The knock down efficiency were assessed by western blot.

### Limulus amebocyte lysate (LAL) assay

The Limulus Amebocyte Lysate (LAL) assay (Xiamen Bioendo Technology; EC64405) was employed to quantify LPS following the manufacturer's instructions. We first injected the mice with LPS, and then all the blood was sucked out and discarded through cardiac blood collection. We then injected a sufficient amount of endotoxin-free DPBS from the hearts of the mice to flush the remaining blood in the vascular circulation as clean as possible. At the end of perfusion, the brain tissue of mice was removed and mixed with endotoxin-free DPBS for grinding. Then brain homogenate extract was collected for LAL assay. At the same time, the last perfusion effluents were drawn from the outlet (right atrial appendage) for LAL assay. All LAL assay reagent and endotoxin-free consumables were supplied by kit.

#### ELISA and LDH assay

Plasma samples from adult mice and cell-free supernatant were examined using TNF-α (ThermoFisher; 88–7324), IL-1α (ThermoFisher; 88–5019), IL-6 (ThermoFisher; 88–701364), or IL-1β (ThermoFisher; 88–7013), ELISA kits. Cell death was assessed via the LDH Cytotoxicity Assay kit (Beyotime Biotechnology).

#### Proximity-ligation assay (PLA)

The experiment of Proximity Ligation Assay kit (Sigma DUO92008) was utilized to reveal the interaction between Casp11 protein and LPS in sections of mouse brain tissue. This unique method allows for the visualization of subcellular localization and protein–protein interactions in situ. Mice were exposed to LPS for 11 h, and 20 μm thick frozen sections of brain tissue were prepared as described earlier. Following fixation with 4% formaldehyde and penetration with TritonX-100, sections were incubated overnight with different kinds of primary antibody pairs for Casp11 (rat monoclonal 17D9, Novus NB120-10454) and LPS (mouse monoclonal 2D7/1, Abcam ab35654). After the primary antibody incubation, the sections were treated with the corresponding PLA probe—coupled oligonucleotide secondary antibody combination (rat MINUS and mouse PLUS for Casp11 and LPS interactions). The Proximity Ligation Assay was executed according to the manufacturer’s instructions, and images were captured by using a Nikon Ni-U microscope.

#### Bone marrow transplantation (BMT)

BMT mice, aged between 5 and 9 weeks, were employed, ensuring the use of sex-matched donor-recipient pairs (all BMT groups represented as donor → recipient). WT donor mice were on the B6.SJL background, and knockouts were on the C57BL/6 background, while recipient mice were on the C57BL/6 background, encompassing both WT and knockout. Bone marrow transplant recipients received 0.5 million Bone marrow cells from donors following irradiation with 12 Gy (administered in split doses of 2 × 6.0 Gy spaced 4 h apart at a rate of 0.5 Gy/min). The recipients were then raised aseptically in an air-laminar flow cabinet and given antibiotic water for 2 weeks. Hematopoietic reconstitution of chimeric mice was detected 4–5 weeks after BMT. Briefly, PBMC were obtained from peripheral blood of recipient mice, and the expressions of CD45.1 and CD45.2 were detected by flow cytometry. Subsequently, the chimeric mice were injected with LPS and the NOR experiment was performed several days after injection.

#### Statistical analysis

We used the Version 9.0 Prism GraphPad and IBM SPSS Statistics 21 for statistical analyses. Data underwent analysis used by One-way or Two-way ANOVA and Repeated Measures ANOVA. p < 0.05 was applied for statistical significance. All experiments were replicated at least three times independently, and all values are expressed as the mean ± SEM.

## Results

### Casp11 promotes cognitive dysfunction after LPS challenge.

**Fig 1:**
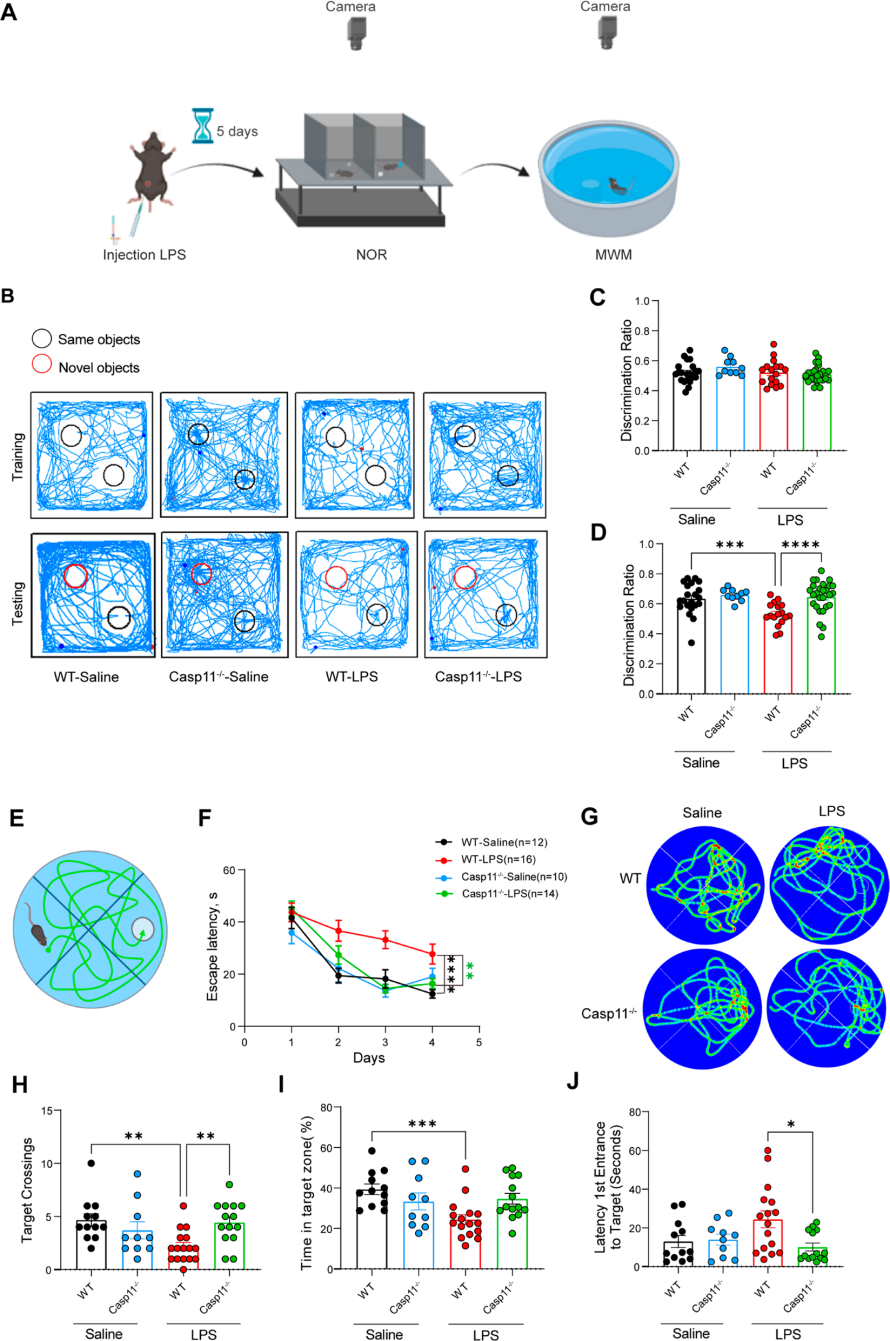
Casp11 promotes cognitive dysfunction after LPS challenge. **A** The strategy of the experiment. Systemic treatments consisted of an intraperitoneal injection of 8 mg/kg LPS or saline. **B**–**D** NOR experiment. WT-Saline (n = 21), Casp11^−/−^-Saline (n = 10), WT-LPS (n = 17), Casp11^−/−^-LPS (n = 27), from four independent experiments. **B** Trajectory tracking of the representative mice. **C** Discrimination ratio in the training period. **D** Discrimination ratio in the testing period. **E**–**J** MWM experiment. WT-Saline (n = 12), Casp11^−/−^-Saline (n = 10), WT-LPS (n = 16), Casp11^−/−^-LPS (n = 14), from three independent experiments. **E** Diagram of the water maze partition. **F** Escape latency in the MWM training task. **G** Trajectory tracking of the representative mice in testing task by the Smart v3.0-Panlab Harvard Apparatus, from three independent experiments with similar results. **H** The number of platform area crossings of mice in testing task. **I** The percentage of time spent in the target quadrant by mice in testing task. **J** The time of first occurrence to the platform area (latency of the 1st entrance to the target) of mice in testing task. The data are expressed as mean ± SEM. *p < 0.05, **p < 0.01, ***p < 0.001, ****p < 0.0001

Cognitive dysfunction caused by infection is an important clinical problem. To elucidate the impact of Casp11 on cognition during infection, we employed an LPS-induced endotoxemia mouse model (Savi et al. 2021). We designed a behavioral experiment to assess the cognitive function of mice (Fig. 1A). The NOR experiment was used to assess the object recognition memory of the mice (a novel object was placed into the red circle). Discrimination ratio presents the proportion of time spent exploring one of the two identical objects. During the training phase of the NOR test, we found that the discrimination ratio of the mice was approximately 0.5, with no significant differences between the groups, meaning that the results would not be biased by the experimental conditions and setup (Fig. 1C). The discrimination ratio of the mice in the WT-LPS group was also approximately 0.5 in the test phase, while the mice in the Casp11^−/−^-LPS and WT-saline groups had discrimination ratios over 0.6 with statistically significant differences (Fig. 1D). The trajectory density visually illustrates the mice's inclination to explore objects (Fig. 1B). This suggests impaired recognition memory for the old object in the WT-LPS group, where mice struggled to recognize the new object. By contrast, akin to WT saline mice, those in the Casp11^−/−^-LPS group exhibited restored cognitive function. In the Morris water maze, LPS-treated mice displayed prolonged escape latency compared to the WT-normal saline group during hidden platform water maze training (Fig. 1F). Notably, the Casp11^−/−^-LPS group exhibited performance akin to the WT-saline group. The LPS-treated mice showed diminished target platform crossings, spent reduced percentages of time in the target quadrant, and experienced increased difficulty locating the hidden platforms. Additionally, the Casp11^−/−^-LPS group closely resembled the WT-saline group (Fig. 1G–J). These findings showed that Casp11 signaling is involved in cognitive impairment of mice after LPS challenge.

### GSDMD enhances cognitive dysfunction in endotoxemic mice.

**Fig 2:**
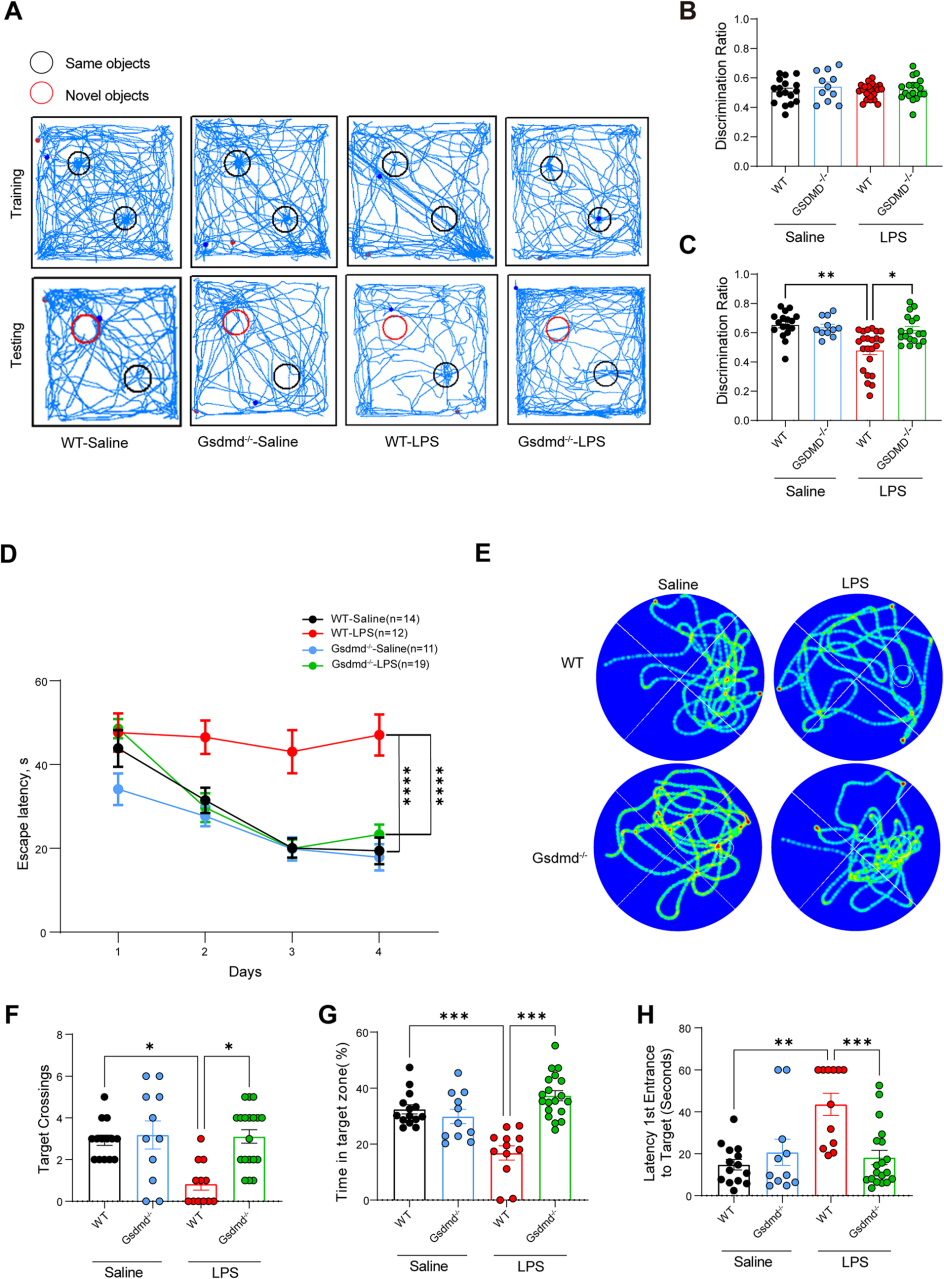
GSDMD enhances cognitive dysfunction in endotoxemic mice. (A-C) NOR experiment. WT-Saline (n = 17), Gsdmd^−/−^-Saline (n = 11), WT-LPS (n = 23), Gsdmd^−/−^-LPS (n = 18), from three independent experiments. **A** Trajectory tracking of representative mice. **B** Discrimination ratio in the training period. **C** Discrimination ratio in the testing period. **D**–**H** MWM experiment. WT-Saline (n = 14), Gsdmd^−/−^-Saline (n = 11), WT-LPS (n = 12), Gsdmd ^−/−^-LPS (n = 19), from three independent experiments. **D** Escape latency in the MWM training task. **E** Trajectory tracking of representative mice in testing task, from three independent experiments with similar results. **F** The number of platform area crossings of mice in testing task. **G** The percentage of time spent in the target quadrant by mice in testing task. **H** The time of first occurrence to the platform area of mice in testing task. The data are expressed as mean ± SEM. *p < 0.05, **p < 0.01, ***p < 0.001, ****p < 0.0001

GSDMD can be cleaved by activated Casp11 (Shi et al. 2015; He et al. 2015; Kayagaki et al. 2015). To determine whether GSDMD was also involved in the formation of cognitive impairment in endotoxemic mice, behavioral tests were performed. During the NOR task, deletion of GSDMD significantly ameliorated the novel object recognition performance in LPS-challenged mice (Fig. 2B, C). Traces of movements in the NOR task showed similar trends (Fig. 2A). Moreover, GSDMD-deficient mice exhibited enhanced performance in the water maze task (Fig. 2D, E), demonstrating more target platform crossings, higher percentages of time spent in the target quadrant, and a faster location finding compared to WT mice (Fig. 2F–H). These findings showed that GSDMD also enhances cognitive dysfunction.

### The effect of the IL-1 cytokine aggravates cognitive dysfunction.

**Fig 3:**
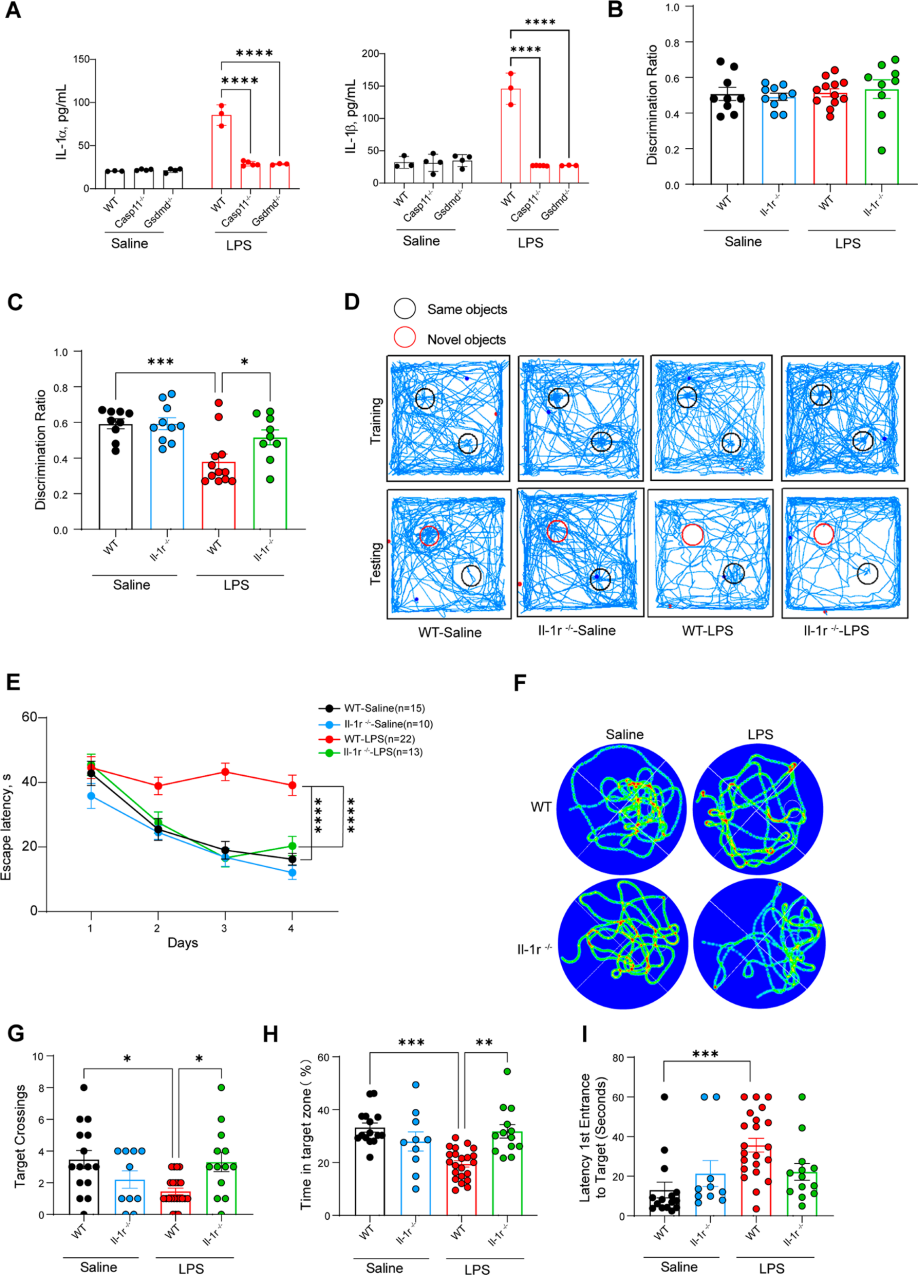
The effect of the IL-1 cytokine aggravates cognitive dysfunction. **A** IL-1α and IL-1β levels in the plasma of mice intraperitoneally challenged with LPS for 16h, n = 3–4 per group. **B**–**D** NOR experiment. WT-Saline (n = 9), Il-1r^−/−^-Saline (n = 10), WT-LPS (n = 12), Il-1r^−/−^-LPS (n = 9), from three independent experiments. **B** Trajectory tracking of representative mice. **B** Discrimination ratio in the training period **C** Discrimination ratio in the testing period. **E**-**I** MWM experiment. WT-Saline (n = 15), Il-1r^−/−^-Saline (n = 10), WT-LPS (n = 22), Il-1r^−/−^-LPS (n = 13), from three independent experiments. **E** Escape latency in the MWM training task. **F** Trajectory tracking of representative mice in testing task, from three independent experiments with similar results. **G** The number of platform area crossings of mice in testing task. **H** The percentage of time spent in the target quadrant by mice in testing task. **I** The time of first occurrence to the platform area of mice in testing task. The data are expressed as mean ± SEM. *p < 0.05, **p < 0.01, ***p < 0.001, ****p < 0.0001

Casp11 and GSDMD signal may mediate the release of inflammatory factors, including IL-1α and IL-1β (Shi et al. 2015). To determine the influence of inflammatory cytokines in more detail, levels of inflammatory cytokines were measured in endotoxemic mice. Casp11 or GSDMD deficiency significantly reduced the release of the inflammatory cytokines IL-1α and IL-1β in the plasma (Fig. 3A). Next, NOR experiment results showed that IL-1R knockout mice performed better than wild-type mice (Fig. 3B–D). Furthermore, the deletion of IL-1r ameliorated the Morris water maze performance in LPS-challenged mice, with shorter escape latency during hidden platform water maze training (Fig. 3E). In the testing period, IL-1r-deficient mice showed more target platform crossings and greater percentages of time spent in the target quadrant and were faster at finding the location than WT mice (Fig. 3F–I). Altogether, these results reveal that inflammatory cytokines make an important contribution to cognitive dysfunction.

### Casp11-GSDMD signaling promotes damage to the hippocampal CA3 region.

**Fig 4:**
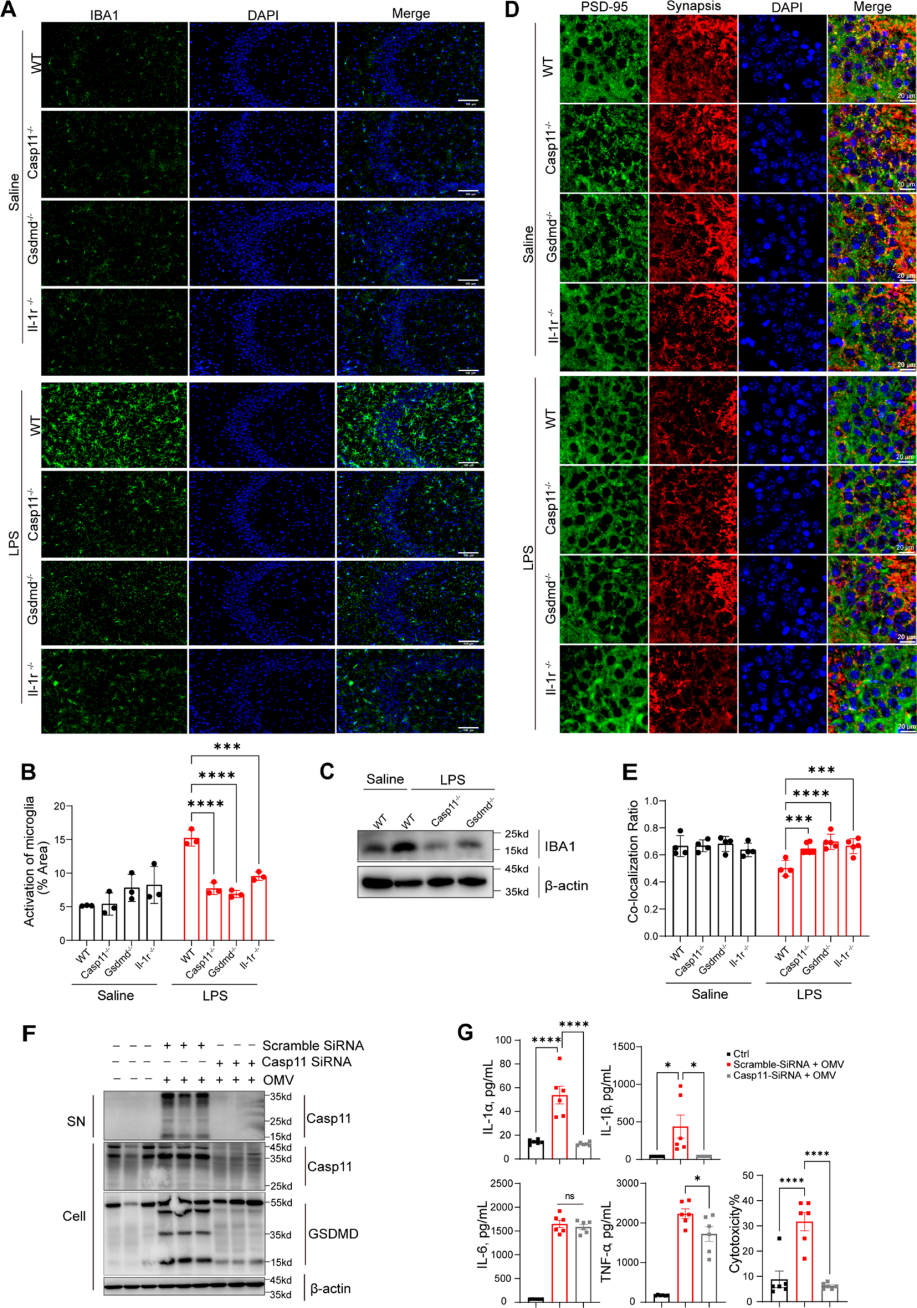
Casp11 and GSDMD promotes damage to the hippocampal CA3 region. **A** Immunofluorescent staining in the hippocampal CA3 region. (IBA1: green, the marker of microglia). Scale bar represents 100μm, n = 3 per group. **B** The fluorescence intensity of microglial activation was quantified by ImageJ software. **C** Immunoblot indicating IBA1 in the hippocampus of mice. **D** Loss of synapses in CA3 as determined by quantification of colocalized pre- and postsynaptic puncta on confocal images. Scale bar represents 20μm, n = 4–6 mice per group. **E** ImageJ was used to determine the number of synaptic colocalized. **F** Immunoblot to detect Casp11, GSDMD in supernatants (SN) or cell lysates (Cell) in BV2 microglia transfected with scrambled siRNA or Casp11-specifific siRNA upon OMV 25μg/mL for 18h. **G** LDH assay for cytotoxicity and ELISA for total IL-6, IL-1α, IL-1β, and TNF-α in cell culture medium. The data are expressed as mean ± SEM. *p < 0.05, **p < 0.01, ***p < 0.001, ****p < 0.0001

The hippocampus, vital for spatial memory, plays a pivotal role in understanding how Casp11 and GSDMD contribute to cognitive dysfunction in sepsis. Our investigation into microglial activation revealed a significant increase in microglial activity in the hippocampal CA3 region of LPS-challenged mice, a response that was restored in Casp11 or GSDMD-deficient mice (Fig. 4A, B). Subsequently, western blot analysis confirmed a notable elevation in IBA1 protein levels in the hippocampus of LPS-exposed groups compared to saline controls, a rise that was mitigated in Casp11 or GSDMD-deficient mice (Fig. 4C). Synapse loss was evident in the hippocampal CA3 regions (Fig. 4D, E). The noteworthy impact of Casp11 and GSDMD warrants attention. In vivo, bacteria-released OMVs were found capable of delivering LPS into mouse macrophages' cytosol, activating Casp11 (Vanaja et al. 2016). Furthermore, to elucidate the effect of Casp11-GSDMD on microglia, immunoblot showed that BV2 microglia exhibited Casp11 activation and GSDMD cleavage, and LDH assay or ELISA showed the release of lactate dehydrogenase and IL1 when treated BV2 cells with E.coli-derived OMVs (Fig. 4F, G). These results suggest that Casp11-GSDMD signaling may play an important role in associated with overactivation of microglia.

### Infection increased BBB permeability and neuroinflammation via Casp11 signaling.

**Fig 5:**
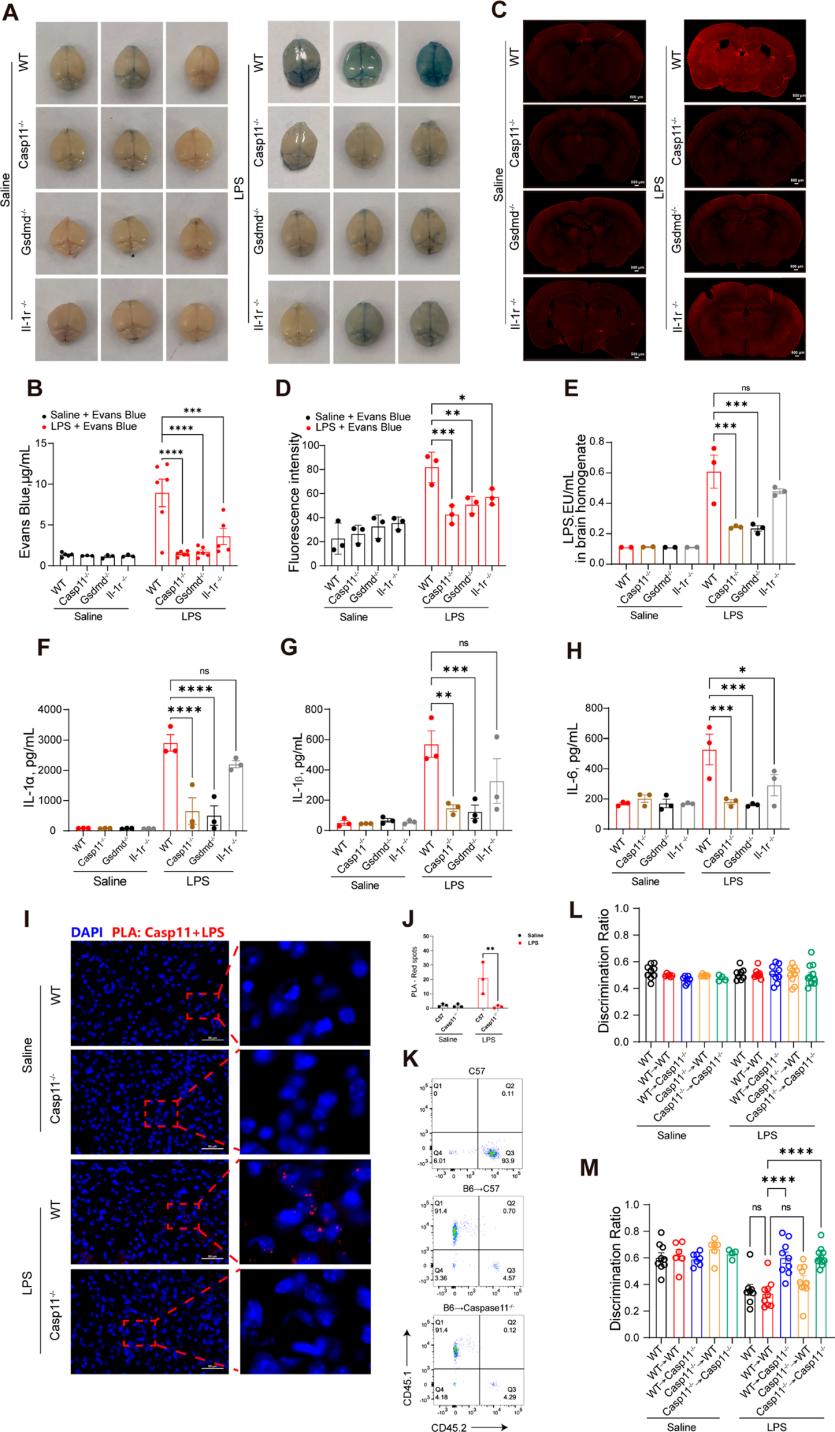
Infection increased BBB permeability and neuroinflammation via Casp11 signaling. **A**-**C** Mice were treated with LPS and 2% Evans blue (2 h, i.v.). **A** Representative photographs of the gross brains of mice, from three independent experiments with similar results. **B** The concentration of Evans blue was expressed as μg Evans blue per mL dimethylformamide. n = 3–6 per group. **C** Graphs of frozen coronal brain sections were acquired from three independent experiments with similar results, scale bar represents 500μm. **D** The fluorescence intensity of Graphs of frozen coronal brain sections were quantified by ImageJ. **E** Limulus Amebocyte Lysate (LAL) assay to detect LPS in brain homogenate after perfusion. **F**–**H** IL-1α, IL1β and IL6 levels in brain homogenate of mice intraperitoneally challenged with LPS, n = 3 per group. **I**-**J** The interaction between Casp11 and LPS were visualized as the red spots by PLA in mouse frozen sections of brain tissue. Scale bar represents 50 μm. **K** The expressions of CD45.1 and CD45.2 in chimeric mice. **L**, **M** NOR experiment of chimeric mice, WT-Saline (n = 9); WT → WT-Saline (n = 6); WT → Casp11^−/−^-Saline (n = 7); Casp11^−/−^ → WT-Saline (n = 6); Casp11^−/−^ → Casp11^−/−^-Saline (n = 4); WT-LPS (n = 8); WT → WT-LPS (n = 9); WT → Casp11^−/−^-LPS (n = 9); Casp11^−/−^ → WT-LPS (n = 10); Casp11^−/−^ → Casp11^−/−^-LPS (n = 11), from three independent experiments. The data are expressed as mean ± SEM. *p < 0.05, **p < 0.01, ***p < 0.001, ****p < 0.0001

There is a special structure between the peripheral circulation and the internal brain environment, named the BBB (Gao and Hernandes 2021). To further determine the role of Casp11-GSDMD signaling, BBB permeability was measured with Evans blue dye (Gibson and Evans 1937). As seen in the gross photographs, the degree of blue staining in Casp11, GSDMD or IL-1r deficient mice was significantly less than that in the WT mice after LPS challenge and similar to that in the saline group (Fig. 5A). Meanwhile, Evans blue permeation quantitative experiments showed the same results as the gross photographs (Fig. 5B). Panoramic scanning images of coronal brain sections of mice showed the lower degree of fluorescence intensity in Casp11, GSDMD or IL-1r deficient mice after LPS challenge (Fig. 5C, D). In addition, elevated LPS levels were detected in brain homogenates when brain barrier leakage occurs after LPS challenge, which was significantly reduced by genetic deletion of Casp11, Gsdmd or IL-1r (Fig. 5E). Meanwhile, the levels of IL1α, IL1β and IL6 in brain homogenates of Casp11 or Gsdmd deficient mice were significantly lower than those of WT mice, it appears that damage to the blood–brain barrier may be associated with (Fig. 5F–H). These results suggest a key link between blood–brain barrier leakage, neuroinflammation and cognitive decline. To investigate the entry of LPS into brain cells and its activation of Casp11 in vivo, we conducted a proximity-ligation assay (PLA), providing a visual representation of the LPS- Casp11 interaction in endotoxemia tissues. PLA results revealed the induction of LPS- Casp11 interaction in the brain tissue of WT mice but not Casp11-deficient mice (I-J), suggesting the entry of LPS into the brain and subsequent Casp11 activation. To discern whether Casp11 in hematopoietic or non-hematopoietic cells contributes to endotoxemia-induced cognitive decline, we generated bone marrow chimeras through bone marrow transplantation (BMT) using WT and Casp11-deficient mice (L-M). NOR test results demonstrated that the deletion of Casp11 in the non-hematopoietic compartment, not the hematopoietic compartment, prevented endotoxemia-induced cognitive decline (M). Thus, non-hematopoietic Casp11 emerges as a crucial player in endotoxemia-induced cognitive decline.

## Discussion

This study unveils a significant contribution of Casp11 and GSDMD to cognitive impairments and spatial memory loss in a murine sepsis model. In Gram-negative sepsis, circulating endotoxin can infiltrate host cell cytoplasm through high-mobility group box 1 protein (HMGB1) or bacterial outer membrane vesicles. This intrusion triggers the activation of cytosolic Casp11, the endotoxin's intracellular receptor (Deng et al. 2018; Vanaja et al. 2016). Activated Casp11 then enzymatically cleaves its downstream substrate GSDMD, producing membrane-pore-forming peptides that aggregate on the cell membrane. This cascade results in the release of numerous inflammatory mediators, including interleukin-1α (IL-1α) and interleukin-1β (IL-1β), along with the induction of lytic cell death.

The Casp11 and GSDMD-dependent functional abnormalities after endotoxin challenge are associated with the pathological changes in the CA3 region of the hippocampus, including cellular structural disruptions and synaptic loss. During the course of studying the underlying mechanisms, we found that Casp11 and GSDMD were required for endotoxin-increased permeability of blood–brain barrier (BBB), which is a vital structure that importantly regulates the exchange of molecules between the brain and the blood stream and provides protection against substance such as certain cytokines that are harmful for the central nerve system. Previous studies have reported changes in BBB permeability in sepsis (Gao and Hernandes 2021; Chung et al. 2020). We show that the endotoxin-induced BBB leakage is mediated by the activation of the Casp11 pathway. Casp11 is highly expressed in endothelial and myeloid cells. As endothelial cells are the major components that constitute BBB, it is conceivable that Casp11 induced endothelial damage or dysfunction might directly increase the permeability of BBB. Previous studies also implicate that immune cell-derived proinflammatory cytokines could also increase the permeability of BBB. Because activation of GSDMD in myeloid cells is associated with the release of interleukin-1 family cytokines, myeloid Casp11 might also contribute to the BBB leakage.

Understanding the mechanisms underlying infection-induced cognitive impairments is of great importance. Our findings shed light on the role of Casp11 and GSDMD in the disruption of the BBB and the subsequent neuroinflammation that contributes to synaptic loss and cognitive dysfunction. Further investigations should focus on elucidating whether and how Casp11 dependent BBB permeability causes neuroinflammation and cognitive decline in sepsis. In conclusion, data in current study demonstrates the involvement of the Casp11 and GSDMD in the breakdown of the blood–brain barrier, the enhance of neuroinflammation, and the loss of synaptic connections in the hippocampus, all of which are associated with the cognitive decline. These findings not only provide scientific evidence and a mechanistic basis for infection-induced persistent cognitive impairments, but also offer potential therapeutic targets for the prevention and treatment of cognitive impairment caused by infection.

## Data Availability

The datasets used and/or analysed during the current study are available from the corresponding author or on reasonable request.
